# Hippocampal PGC-1α-mediated positive effects on parvalbumin interneurons are required for the antidepressant effects of running exercise

**DOI:** 10.1038/s41398-021-01339-1

**Published:** 2021-04-15

**Authors:** Jin Wang, Jing Tang, Xin Liang, Yanmin Luo, Peilin Zhu, Yue Li, Kai Xiao, Lin Jiang, Hao Yang, Yuhan Xie, Lei Zhang, Yuhui Deng, Jing Li, Yong Tang

**Affiliations:** 1 Laboratory of Stem Cells and Tissue Engineering, Faculty of Basic Medical Sciences, Chongqing Medical University, 400016 Chongqing, People’s Republic of China; 2 Department of Histology and Embryology, Faculty of Basic Medical Sciences, Chongqing Medical University, 400016 Chongqing, People’s Republic of China; 3 Department of Pathophysiology, Faculty of Basic Medical Sciences, Chongqing Medical University, 400016 Chongqing, People’s Republic of China; 4 Department of Physiology, Faculty of Basic Medical Sciences, Chongqing Medical University, 400016 Chongqing, People’s Republic of China; 5 Lab Teaching & Management Center, Chongqing Medical University, 400016 Chongqing, People’s Republic of China

**Keywords:** Depression, Neuroscience

## Abstract

Running exercise was shown to have a positive effect on depressive-like symptoms in many studies, but the underlying mechanism of running exercise in the treatment of depression has not been determined. Parvalbumin-positive interneurons (PV^+^ interneurons), a main subtype of GABA neurons, were shown to be decreased in the brain during the depression. PGC-1α, a molecule that is strongly related to running exercise, was shown to regulate PV^+^ interneurons. In the present study, we found that running exercise increased the expression of PGC-1α in the hippocampus of depressed mice. Adult male mice with PGC-1α gene silencing in the hippocampus ran on a treadmill for 4 weeks. Then, depression-like behavior was evaluated by the behavioral tests, and the PV^+^ interneurons in the hippocampus were investigated. We found that running exercise could not improve depressive-like symptoms or increase the gene expression of PV because of the lack of PGC-1α in the hippocampus. Moreover, a lack of PGC-1α in the hippocampus decreased the number and activity of PV^+^ interneurons in the CA3 subfield of the hippocampus, and running exercise could not reverse the pathological changes because of the lack of PGC-1α. The present study demonstrated that running exercise regulates PV^+^ interneurons through PGC-1α in the hippocampus of mice to reverse depressive-like behaviors. These data indicated that hippocampal PGC-1α-mediated positive effects on parvalbumin interneurons are required for the antidepressant actions of running exercise. Our results will help elucidate the antidepressant mechanism of running exercise and identify new targets for antidepressant treatment.

## Introduction

Depression is a common mood disorder that has become a major threat to social stability and human development due to its high incidence, high recurrence rate, and high disability rate^1,2^. However, existing antidepressants have some obvious disadvantages. For example, fluoxetine, a classic antidepressant, has a slow onset and a low response rate^3^, and ketamine, a rapid antidepressant, is usually short-lasting and has a risk of abuse^4^. Running exercise is a simple and feasible behavioral intervention for the treatment of depression and has been proved to be an effective treatment for depression and has been widely accepted as an effective therapy^5^. Clinical studies have found that moderate and vigorous running exercise relieves depressive symptoms in major depressive disorder (MDD)^6^ and undertaking regular leisure-time exercise was associated with reduced the risk of future depression in adults^7^. Animal studies also demonstrated that running exercise could improve depression-like symptoms of different depressed model rats and mice^8–10^. Although running exercise is effective in treating depression, the underlying mechanism is unknown.

Many studies have shown that stress can disrupt the function of GABA, which plays a major role in the potential inhibitory deficits of depression^11,12^. GABA interneurons can be divided into several subtypes, among which parvalbumin-positive interneurons (PV^+^ interneurons) account for approximately 40%^13^. PV^+^ interneurons represent a subgroup of GABAergic interneurons with high metabolism and high electrical activity, which are important markers of the central nervous inhibitory system^14^. The hippocampus is closely related to emotional regulation and is an important stress-susceptible area associated with depression^15,16^. PV^+^ interneurons in the hippocampus are very sensitive to various types of damage, such as sustained chronic stress, ischemia and hypoxia, which lead to damage and dysfunction of PV^+^ interneuron^17,18^. As PV^+^ interneurons have a significant regulatory function in the hippocampus, they have been widely investigated in studies of depression. Several animal studies have reported that the number or density of PV^+^ interneurons in the hippocampus is significantly decreased in various animal models of depression^19–21^, indicating that PV^+^ interneurons are involved in the pathogenesis of depression. Running exercise has been proven to be beneficial to PV^+^ interneurons in hippocampus. Arriaga et al. observed the CA1 subfield of the hippocampus in male and female mice by two-photon calcium imaging in vivo to characterize the relationship between interneuron activity and movement and found that the activity of most PV^+^ interneurons was positively correlated with motor activity^22^. Nguyen et al. found that the numbers of PV^+^ interneurons in the hippocampal CA1 and CA2-3 subfields were increased after 30 days of running exercise in adult male rats^23^. Gomes et al. found that the expression of PV protein in the hippocampus and the numbers of PV^+^ interneurons in the CA1 and CA2-3 subfields of male rats were increased significantly after running exercise from 21 days to 60 days of age^24^. These animal studies indicated that physical exercise could have positive effects on PV^+^ interneurons in the hippocampus. However, no studies have investigated whether running exercise relieves depressive-like behaviors by affecting PV^+^ interneurons in the hippocampus.

If running exercise can relieve depression by affecting hippocampal PV^+^ interneurons, what might be its molecular mechanism? Peroxisome proliferator-activated receptor γ coactivator 1α (PGC-1α) proteins are nuclear transcription-assisted activators that play a key role in regulating the activities of multiple nuclear and non-nuclear receptors. The expression of PGC-1α is moderate in brain tissue, and this molecule is expressed in brain areas that are related to depression, such as the hippocampus^25^. Treadmill running was shown to increase both the mRNA expression and protein expression of PGC-1α in the hippocampus of healthy rodents^26,27^. In addition, some rodent studies demonstrated that running exercise could exert neuroprotective effects through PGC-1α in the hippocampus^28,29^. Therefore, we speculated that running exercise might improve depressive-like symptoms by affecting hippocampal PGC-1α, but no study has examined this hypothesis. Moreover, PGC-1α has an important regulatory effect on PV^+^ interneurons. Previous studies have suggested that the expression of PGC-1α could affect the expression of PV protein and the function of PV^+^ interneurons. The PV protein expression in PGC-1α+/− and PGC-1α−/− mouse brains was significantly decreased, which affected the function of PV^+^ interneurons^30^, Furthermore, overexpression of PGC-1α in cell culture was sufficient to induce PV protein expression, demonstrating that PGC-1α is necessary for the expression of PV on neurons in the brain^30^. In addition, Lucas et al. found that the PV^+^ interneuron-specific genes SYT2, CPLX1, and NEFH had a similar trend of expression as PGC-1α during development^31^. Under conditional deletion of PGC-1α in the mouse cortex, the transcriptional expression of these genes in PV^+^ interneurons was significantly reduced^31^. Therefore, we speculated that running exercise might protect PV^+^ interneurons through PGC-1α in the hippocampus to reverse depressive-like behaviors.

In the present study, C57BL/6 mice underwent PGC-1α gene silencing in the hippocampus, followed by treadmill running. Then, the effects of treadmill running on PV^+^ interneurons in the hippocampus of the mice were investigated with stereological methods, immunohistochemistry, western blotting, and qRT-PCR. Our study found that silencing hippocampal PGC-1α could induce depressive-like behavior and a decrease in PV^+^ interneurons in the hippocampus of mice, but running exercise could not reverse these changes when PGC-1α was knocked down in the hippocampus. Our results provide important evidence for the antidepressant mechanism of running exercise.

## Methods

### Animals

Six- to eight-week-old male C57BL/6 mice (Chongqing Medical University, Chongqing, China) were housed under a 12-h light/12-h dark cycle at a constant temperature (22 °C) with free access to food and water for 2 weeks to habituate to the housing conditions. During the animal experiment, the animals in each group were treated by the investigators without blinding. No animals were excluded from the current study. All procedures of the experiment were conducted in accordance with the National Institutes of Health Guide for the Care and Use of Laboratory Animals.

### Chronic unpredictable stress (CUS)

Mice were randomly divided into the control group (*n* = 18) and the CUS group (*n* = 42). The CUS group mice exposed to CUS stimulation for 5 weeks. These CUS group mice were subjected to a sequence of 14 different stressors^32,33^. After 5 weeks of CUS stimulation, 42 mice in the CUS group were randomly divided into a CUS Standard group (*n* = 20) and a CUS + Running group (*n* = 22). The control group was not given any intervention during the establishment of the model for 5 weeks.

### Stereotactic injection of adeno-associated virus (AAV) targeting PGC-1α

Mice were randomly divided into the AAV-GFP group (*n* = 30) and AAV-PGC-1α group (*n* = 31). The mice in the AAV-GFP group were injected with HBAAV2/9-GFP (siRNA:TTCTCCGAACGTGTCACGTAA) and the mice in the AAV-PGC-1α group were injected with HBAAV2/9-m-Ppargc1a shRNA2-GFP (AAV-PGC-1α-shRNA) (siRNA:TAACTATGCAGACCTAGATAC) in the hippocampus with a stereotactic injection technique. The virus was generated to target the PGC-1α gene through a doxycycline-inducible shRNA. After 4 weeks of recovery, half of the mice in the AAV-GFP group and the AAV-PGC-1α group were randomly chosen to constitute the AAV-GFP + RN group (*n* = 15) and the AAV-PGC-1α + RN group (*n* = 16) to accept treadmill running for 4 weeks.

### Treadmill running

The program of treadmill running based on previous research^34^ was detailed described in the Supplementary.

### Behavioral tests

After a series of interventions, depression-like behavior of mice was evaluated by behavioral tests such as sucrose preference test (SPT)^35^, forced swimming test (FST)^36^ and tail suspension test (TST)^37^. The additional details are provided in the Supplementary.

### Immunohistochemistry and stereological cell counting

During the following processes, all the experiments and data analyses were performed blind to treatment conditions. After the preparation of the hippocampus tissue (Supplement), sections were chosen and immunoreacted with anti-PV antibody for the stereologic analyses of the total numbers of PV^+^ interneuron^38^ and PV/cFos antibodies for the analyses of the activity PV^+^ interneuron. Specific operations and quantification are available in Supplementary.

### Quantitative real-time PCR, Western blotting, and enzyme-linked immunosorbent assays (ELISAs)

Specific operations and oligonucleotide primers specific for mouse are listed in the supplementary.

### Statistical analyses

The statistical analyses were conducted using SPSS 23.0 statistical software and are described in detail in the Supplementary. The sample size of each experiment was selected based on previous experience, in order to detect at least *p* < 0.05 in different tests.

## Result

### Running exercise alleviated anhedonia induced by CUS

**Fig. 1:**
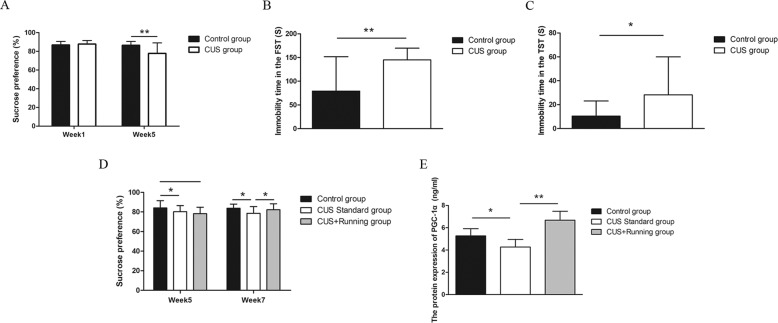
The effects of running exercise on the depressive-like behaviors and protein expression of PGC-1α in the hippocampus of mice in different groups. **A**–**C** The sucrose preference changes, immobility time in the FST and TST (mean ± SD) of mice in the control group (*n* = 18) and the CUS group (*n* = 42) for the first five weeks. * indicates *p* < 0.05, and ** indicates *p* < 0.001. **D** The sucrose preference changes (mean ± SD) of mice among the control group (*n* = 18), the CUS Standard group (*n* = 20), and the CUS + Running group (*n* = 22) for the last two weeks. * indicates *p* < 0.05. **E** The protein expression of PGC-1α in the hippocampus of the mice in the control group (*n* = 5), the CUS Standard group (*n* = 5) and the CUS + Running group (*n* = 5) (mean ± SD).* indicates *p* < 0.05, ** indicates *p* < 0.001.

At the beginning of CUS, the percentage of sucrose preference in the control group did not differ from that in the CUS group (*p* = 0.36). At the 5th week, the percentage of sucrose preference in the CUS group was significantly lower than that in the control group (*p* < 0.001) (Fig. 1A). Moreover, the immobility time in the FST (*p* < 0.001) and TST (*p* = 0.005) of the CUS group was significantly longer than that in the control group (Fig. 1B, C). After 2 weeks of running exercise, the percentage of sucrose preference in the CUS Standard group was significantly lower than that in the control group (*p* = 0.008) and the CUS + Running group (*p* = 0.045) (Fig. 1D).

### Running exercise alleviated the decrease of PGC-1α induced by CUS

We detected the protein expression of PGC-1α in the hippocampus of the mice in the control group, the CUS Standard group and the CUS + Running group. The protein expression of PGC-1α in the CUS Standard group was significantly lower than that in the control group (*p* = 0.045). Moreover, the protein expression of PGC-1α in the CUS Standard group was significantly lower than that in the CUS + Running group after 2 weeks of running exercise (*p* < 0.001) (Fig. 1E).

### Running exercise could not alleviate depressive-like symptoms of the hippocampal PGC-1α-silenced mice

**Fig. 2:**
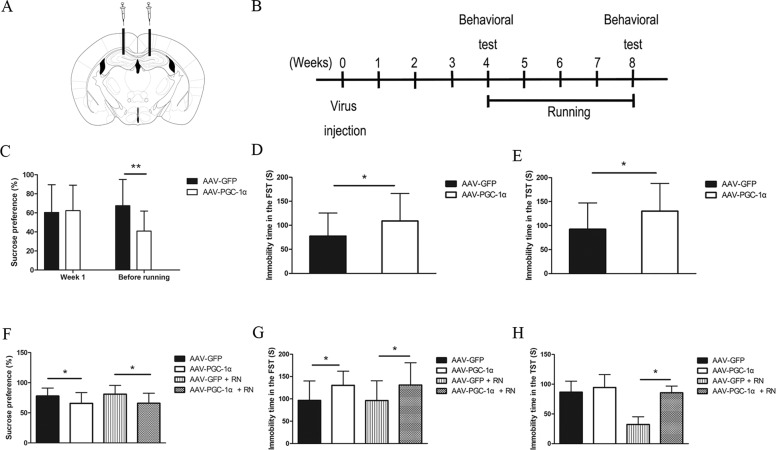
Comparisons of the behavioral test results of mice in different groups. **A** Schematic showing the positions of adeno-associated virus injection in hippocampus of mice. **B** Schematic showing behavior experiment timeline. The behavioral tests include SPT, FST and TST. **C**–**E** The sucrose preference and immobility time in the FST and TST (mean ± SD) of mice in AAV-GFP group (*n* = 30) and the AAV-PGC-1α group (*n* = 31) before running exercise. * indicates *p* < 0.05, and ** indicates *p* < 0.001. **F**–**H** The sucrose preference and immobility time in the FST and TST (mean ± SD) of mice in the AAV-GFP group (*n* = 15) and AAV-PGC-1α group (*n* = 15), AAV-GFP + RN group (*n* = 15) and AAV-PGC-1α + RN group (*n* = 16) after running exercise. * indicates *p* < 0.05.

After the mice were injected with adeno-associated virus in the hippocampus with a stereotactic injection technique (Fig. 2A), the intervention and behavioral tests were carried out according to the schedule (Fig. 2B). We detected the percentage of sucrose preference in the AAV-GFP group and AAV-PGC-1α group immediately after virus injection. The percentage of sucrose preference in the AAV-GFP group did not differ from that in the AAV-PGC-1α group (*p* = 0.753). After 4 weeks of viral transfection, the percentage of sucrose preference in the AAV-PGC-1α group was significantly lower than that in the AAV-GFP group (*p* < 0.001)(Fig. 2C). Furthermore, the immobility time in the FST (*p* = 0.024) and TST (*p* = 0.013) of the AAV-PGC-1α group was significantly longer than that of the AAV-GFP group (Fig. 2D, E). Then, the mice in the AAV-GFP + RN group and the AAV-PGC-1α + RN group underwent treadmill running for 4 weeks. The percentage of sucrose preference in the AAV-PGC-1α group was significantly lower than that in the AAV-GFP group (*p* = 0.038) and the percentage of sucrose preference in the AAV-PGC-1α + RN group was significantly lower than that in the AAV-GFP + RN group (*p* = 0.012). However, there was no difference between the AAV-PGC-1α group and the AAV-PGC-1α + RN group in the percentage of sucrose preference (*p* = 0.982) (Fig. 2F). The immobility time in the FST of the AAV-PGC-1α group was significantly longer than that of the AAV-GFP group (*p* = 0.045) and the immobility time in the FST (*p* = 0.032) and TST (*p* = 0.028) in the AAV-PGC-1α + RN group was significantly longer than that of the AAV-GFP + RN group. However, there was no difference between the AAV-PGC-1α group and the AAV-PGC-1α + RN group in immobility time in the FST (*p* = 0.964) and TST (*p* = 0.999) (Fig. 2G, H).

### Running exercise could not alleviate the decrease in the gene and protein expression of PV induced by hippocampal PGC-1α silencing

**Fig. 3:**
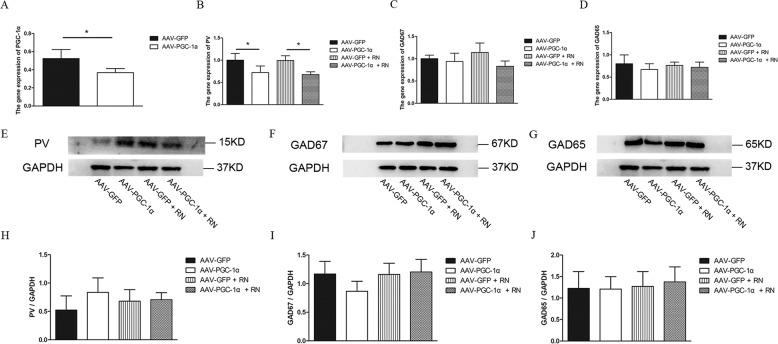
Comparisons of the relative gene and protein expression results in the hippocampus of mice in different groups. **A** The gene expression of PGC-1α in the AAV-GFP group (*n* = 4) and the AAV-PGC-1α group (*n* = 4) (mean ± SD). * indicates *p* < 0.05. **B**–**D** The gene expression of PV, GAD65 and GAD67 in the AAV-GFP group (*n* = 4), AAV-PGC-1α group (*n* = 4), AAV-GFP + RN group (*n* = 4) and AAV-PGC-1α + RN group (*n* = 3) (mean ± SD). * indicates *p* < 0.05. **E**–**G** The protein expression levels of PV, GAD67, and GAD65 in the hippocampus of mice in the AAV-GFP group (*n* = 5), AAV-PGC-1α group (*n* = 5), AAV-GFP + RN group (*n* = 5), and AAV-PGC-1α + RN group (*n* = 5) were detected using western blots. **H**–**J** Semiquantitative analyses of the protein levels of PV, GAD67, and GAD65 in the hippocampus of mice in the AAV-GFP group (*n* = 5), AAV-PGC-1α group (*n* = 5), AAV-GFP + RN group (*n* = 5), and AAV-PGC-1α + RN group (*n* = 5) (mean ± SD).

To assess the effect of virus injection and running exercise after the virus injection on the gene and protein expression of PV, we used RT-PCR and western blotting to detect the expression of PV in the hippocampus in each group of mice. First, we detected the gene expression of PGC-1α at the 4th week after the virus injection, and PGC-1α expression in the AAV-PGC-1α group was significantly lower than that in the AAV-GFP group (*p* = 0.031)(Fig. 3A). After 4 weeks of running exercise, the gene expression of PV in the AAV-PGC-1α group was significantly lower than that in the AAV-GFP group (*p* = 0.01), and the gene expression of PV in the AAV-PGC-1α + RN group was significantly lower than that in the AAV-GFP + RN group (*p* = 0.007). However, there was no difference between the AAV-PGC-1α group and the AAV-PGC-1α + RN group in the gene expression of PV (*p* = 0.66) (Fig. 3B). In addition, the expression levels of genes related to GABA, such as GAD65 and GAD67, were not different among the four groups after running exercise (*p* > 0.05) (Fig. 3C, D). The western blotting results indicated that the protein expression levels of PV, GAD65 and GAD67 were not significantly changed among the 4 groups after virus injection and running exercise (*p* > 0.05) (Fig. 3E–J).

### Running exercise could not alleviate the decrease of the activity of PV^+^ interneurons induced by hippocampal PGC-1α silencing

**Fig. 4:**
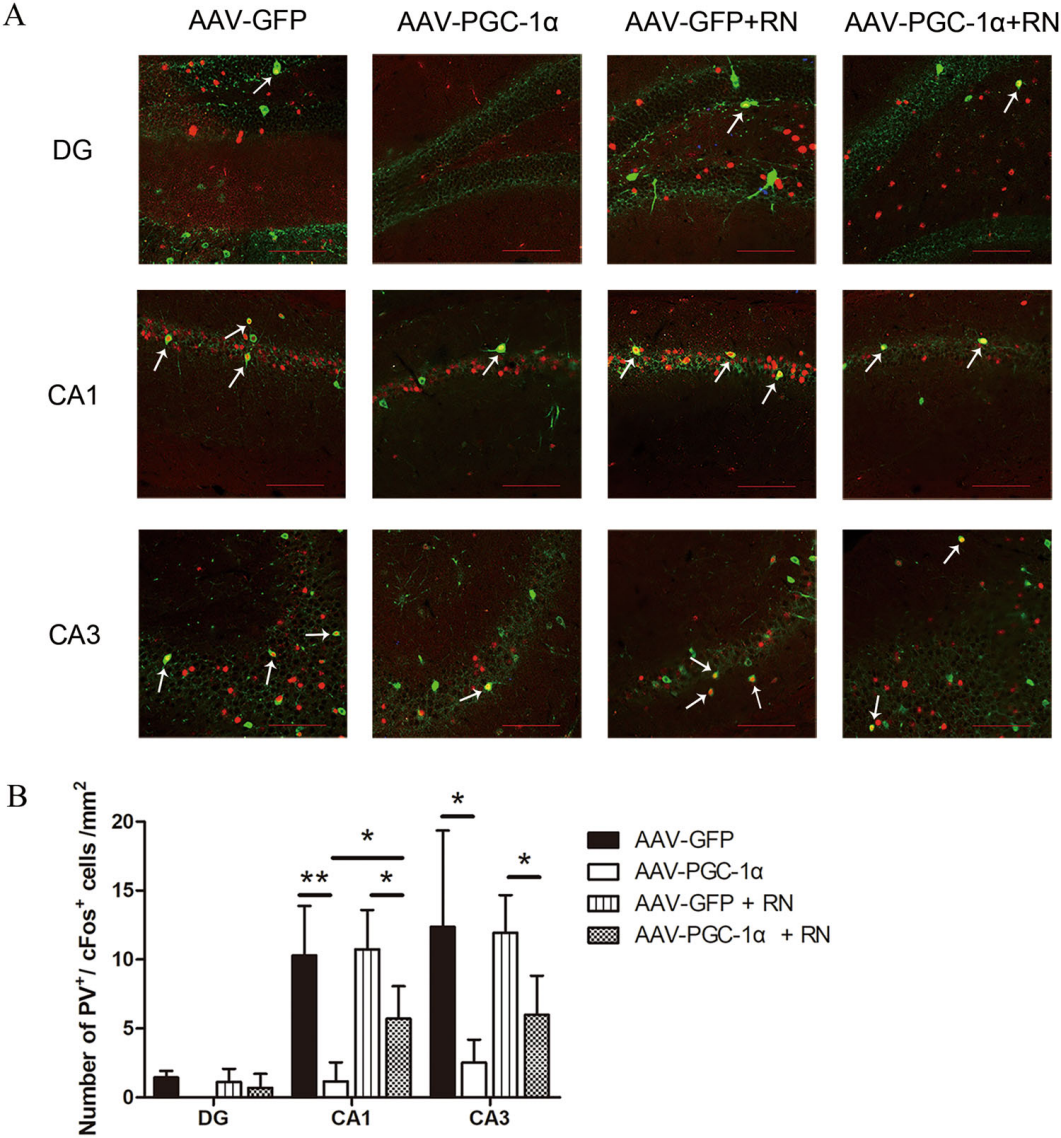
Comparisons of the cFos^+^/PV^+^ cells in the DG, CA1, and CA3 subfields of the hippocampus of the mice in different groups. **A** Representative pictures of immunofluorescence staining with anti-PV antibody and anti-cFos antibody in the hippocampus in the four groups. The white arrows show the cFos^+^/PV^+^ cells. PV: green, c-Fos: red. Scale bar = 100 μm. **B** Quantification of the cFos/PV cell number in the hippocampus in the AAV-GFP group (*n* = 4), AAV-PGC-1α group (*n* = 5), AAV-GFP + RN group (*n* = 4) and AAV-PGC-1α + RN group (*n* = 5) of mice after immunofluorescence staining (mean ± SD). * indicates *p* < 0.05, and ** indicates *p* < 0.001.

Silencing PGC-1α in the hippocampus may lead to functional changes in neurons, but whether functional changes are involved in the occurrence of depression or whether running exercise can protect against depressive symptoms after virus injection is unclear. CFos expression was evaluated as a measure of functional activity, and many studies demonstrated that increased neuronal activity could induce cFos expression^39^, and it has became the most widely used tool to delineate individual neurons^40^. We used double immunohistology to label the cFos/PV neurons so that we could determine whether the activity of PV^+^ interneurons in the hippocampus of the mice changed after virus injection and running exercise. The results demonstrated that the cFos^+^/PV^+^ cells in the CA1 (*p* < 0.001) and CA3 (*p* = 0.002) subfields of the hippocampus of mice in AAV-PGC-1α group were significantly decreased compared with those in the AAV-GFP group, and the cFos^+^/PV^+^ cells in the CA1 (*p* = 0.011) and CA3 (*p* = 0.039) subfields in the AAV-PGC-1α + RN group were significantly decreased compared with those in the AAV-GFP + RN group. The cFos^+^/PV^+^ cells in the CA1 subfield of hippocampus of mice in AAV-PGC-1α group were significantly decreased compared with those in the AAV-PGC-1α + RN group (*p* = 0.015). However, there was no difference between the AAV-PGC-1α group and the AAV-PGC-1α + RN group in the cFos^+^/PV^+^ cells in CA3 subfield of hippocampus (*p* = 0.179). The cFos^+^/PV^+^ cells in the DG subfield of hippocampus showed no significant change among the 4 groups after virus injection and running exercise (*p* > 0.05) (Fig. 4).

### Running exercise could not alleviate the decrease in the number of PV^+^ interneurons induced by PGC-1α silencing

**Fig. 5:**
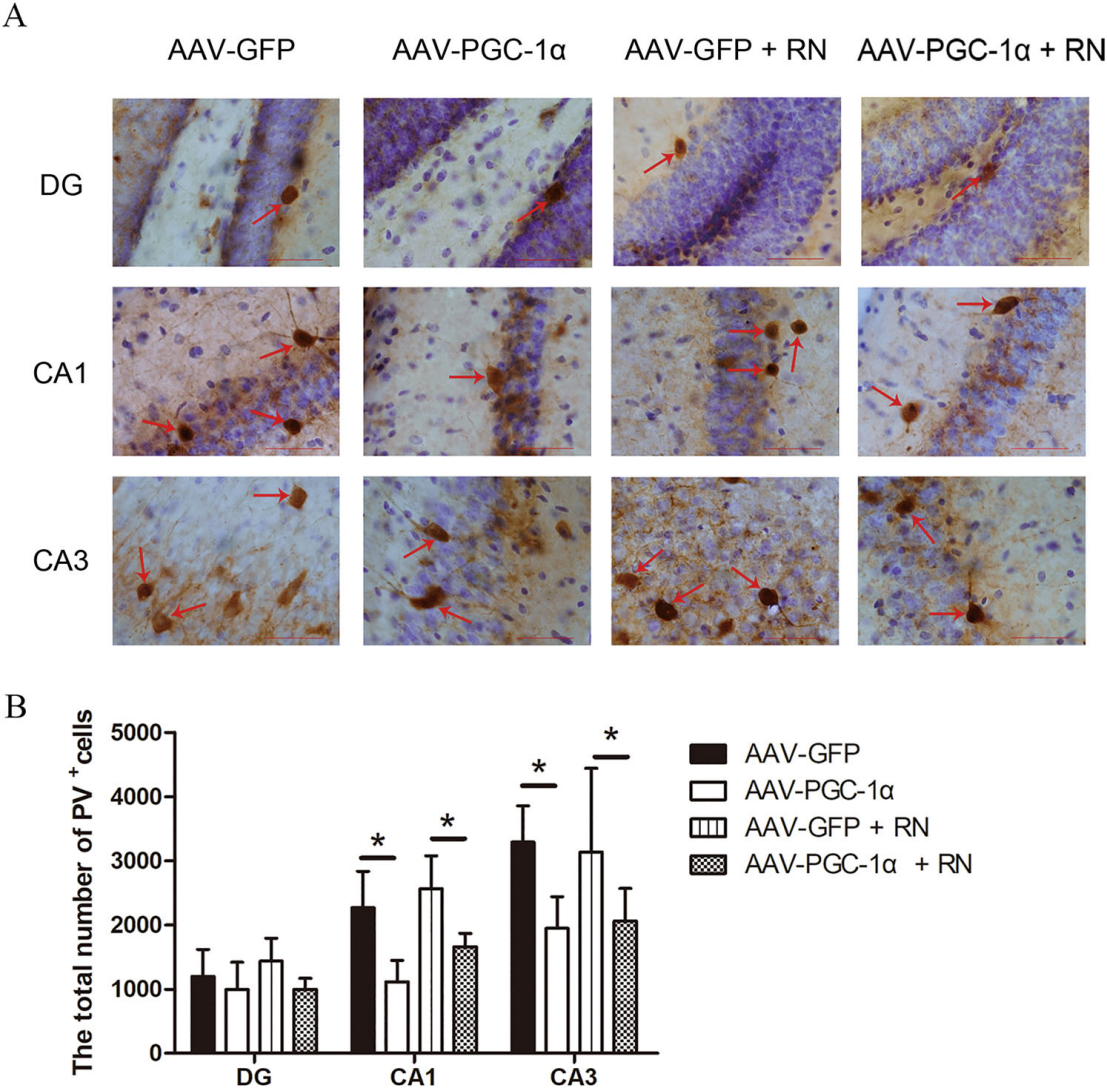
Comparisons of the number of PV^+^ interneurons in the DG, CA1, and CA3 subfields of the hippocampus of the mice in different groups. **A** Representative pictures of immunohistochemical staining with anti-PV antibody in the hippocampus in the four groups of mice. The red arrows show the PV^+^ interneurons. Scale bar = 40 μm. **B** Quantification of PV^+^ interneuron number in the hippocampus in the AAV-GFP group (*n* = 5), AAV-PGC-1α group (*n* = 5), AAV-GFP + RN group (*n* = 5) and AAV-PGC-1α + RN group (*n* = 5) of mice after immunohistochemistry staining (mean ± SD). * indicates *p* < 0.05.

Using the stereological method and immunohistochemic technique, we accurately estimated the total number of PV^+^ interneurons in different subfields of the hippocampus of the mice in the four groups. The number of PV^+^ interneurons in the CA1 (*p* = 0.001) and CA3 (*p* = 0.017) subfields of hippocampus of mice in AAV-PGC-1α group was significantly decreased compared with that in the AAV-GFP group, and the number of PV^+^ interneurons in the CA1 (*p* = 0.004) and CA3 (*p* = 0.047) subfields in AAV-PGC-1α + RN group was significantly decreased compared with that in the AAV-GFP + RN group. However, there was no difference between the AAV-PGC-1α group and the AAV-PGC-1α + RN group in the number of PV^+^ interneurons in the CA1 (*p* = 0.061) and CA3 (*p* = 0.827) subfields of hippocampus. The numbers of PV^+^ interneurons in the DG subfield of hippocampus showed no significant change among the four groups after virus injection and running exercise (*p* > 0.05) (Fig. 5).

## Discussion

The hippocampus is an important brain region related to depression^15,16^. PV^+^ interneurons are an important class of inhibitory interneurons. Some studies have found that PV^+^ interneurons in the hippocampus of individuals with depression have changed^19,20^. The hippocampal structure includes the hippocampal gyrus (CA1, CA3, etc.) and the dentate gyrus (DG). Different subfields of the hippocampus have different functions^41–46^. Filipović et al. found that the PV^+^ interneurons in hippocampal CA1, CA3, and DG were significantly decreased in adult male rats after 21 days of social isolation^20^. Csabai et al. used CMS model rats and found that stress could reduce the density of PV^+^ interneurons in CA1 subfield of hippocampus^21^. The above studies indicated that PV^+^ interneurons were involved in the pathogenesis of depression in the hippocampus. What is the underlying molecular mechanism? In the present study, we found that the protein expression of PGC-1α in the CUS Standard group was significantly lower than that in the control group. We hypothesized that the decrease in PGC-1α induced PV^+^ interneurons changes. To assess this hypothesis, we characterized PGC-1α expression in the hippocampus to study the changes in PV^+^ interneurons in mice. Behavioral tests showed that mice presented a series of depressive-like symptoms after recombinant AAV injection. RT-PCR results indicated that the gene expression levels of PGC-1α and PV in the AAV-PGC-1α group were significantly lower than those in the AAV-GFP group. The results suggested that the lack of PGC-1α could cause depressive-like symptoms in mice and reduce the gene expression of PV in the hippocampus. Does the lack of PGC-1α in the hippocampus induce depressive-like behaviors by affecting PV^+^ interneurons in the hippocampus? To explore this issue, we investigated the activity and number of PV^+^ interneurons in the hippocampus of mice after PGC-1α silencing. We used three-dimensional stereological methods to quantify the total number of PV^+^ interneurons in different subfields of the hippocampus in the mice after PGC-1α silencing. In our study, the total numbers of PV^+^ interneuronsin the CA1 and CA3 subfields in the hippocampus of the mice in the AAV-PGC-1α group were significantly decreased compared to those in the AAV-GFP group. The same trend was found in the activity of PV^+^ interneurons in the CA1 and CA3 subfields of the hippocampus, which are represented by cFos^+^/PV^+^ cells. Therefore, it is reasonable to believe that the cause of depressive-like symptoms in mice might be the change in PV^+^ interneurons in the hippocampus induced by the lack of PGC-1α. Using the unbiased stereological method, we provided the first direct evidence that PGC-1α in the mouse hippocampus affects PV^+^ interneurons in the hippocampus and induces depressive-like behaviors.

Running exercise is a simple and feasible behavioral intervention for depression, it has been proven to be an effective treatment for depression in clinical studies and rodent studies^7,10,47^ but the exact underlying mechanism is unclear. Although many studies have affirmed the positive effects of running exercise on PV^+^ interneurons in healthy animals, there remains a lack of evidence regarding running exercise improving depressive-like symptoms by protecting PV^+^ interneurons and the mechanism of running exercise acting on PV^+^ interneurons. In the current study, we found that 2 weeks of running exercise significantly reversed anhedonia and increased the protein expression of PGC-1α in the CUS standard group. Because PGC-1α is closely related to PV^+^ interneurons, we hypothesized that running exercise regulates PV^+^ interneurons through PGC-1α to improve depressive-like symptoms. To confirm this hypothesis, we conducted a 4-week running intervention in mice with hippocampal PGC-1α silencing. We found that running exercise could not reverse the decrease in the percentage of sucrose preference and the extension of immobility time in the TST and FST caused by PGC-1α silencing. After 4 weeks of running exercise, the gene expression of PV in the AAV-PGC-1α + RN group was significantly lower than that in the AAV-GFP + RN group. As expected, there was no difference between the AAV-PGC-1α group and the AAV-PGC-1α + RN group in the gene expression of PV. However, the protein expression of PV was not different among the four groups. These results indicated that the protein expression and mRNA expression of PV were not consistent. We considered the following reasons. First, mRNA expression could not represent protein expression totally. Schwanhüusser et al. determined that only approximately 40% of the variance in protein levels between different proteins could be explained by mRNA levels^48^. Second, the cellular mRNA concentration is substantially lower than the protein concentration^48,49^. The intracellular protein concentrations were controlled in a relatively stable range in mammalian cells, while the total mRNA level in a cell can vary significantly as a function of cellular state^50^. Therefore, it was easier to detect the change in PV gene expression than the change in PV protein expression. Third, the variation in protein synthesis rates was the major determinant of absolute protein number variation in mammalian cells. That is, post-transcriptional regulation contributes substantially more to protein level changes than immediate changes induced by mRNA^51^. Since the gene expression of PV can determine the function of PV^+^ interneuron to some extent^52,53^, we confirmed that the lack of PGC-1α at the gene transcription level hindered the recovery of PV^+^ interneurons by running exercise, which led to the failure of running exercise in the treatment of depressive-like symptoms.

In the present study, we detected the expression of GABA-related genes, such as GAD65 and GAD67^54^. The RT-PCR results in our study suggested that there was no difference among the four groups after running exercise or the western blotting result. The results suggested that a lack of PGC-1α induced depressive-like symptoms in mice and running exercise improved depressive-like symptoms through the PV, a subtype of GABA. Thus, our study found for the first time that running exercise can improve the depressive-like symptoms of mice by regulating gene expression of PV in hippocampus through PGC-1α, which provided new structural evidence for the antidepressant effects of running exercise.

Our current study indicated that running exercise could not restore the activity of PV^+^ interneurons after silencing PGC-1α in the hippocampus of mice. Previous studies have shown that different stimuli can influence the expression of cFos, a functional marker of activated neurons^39,40,55,56^ in different brain areas of mice after stress intervention^57,58^. However, these researchers did not detect the depressive-like symptoms in mice using behavioral tests, so their conclusions were not related to depression. In our study, counting was performed on the basis of a series of behavioral tests for depression to ensure that our results represented changes in the hippocampal PV activity of depressed mice. We found that the cFos^+^/PV^+^ cells in the CA1 and CA3 subfields of the hippocampus of the mice in the AAV-PGC-1α group were significantly decreased compared with those in the AAV-GFP group, and the cFos^+^/PV^+^ cells in the CA1 and CA3 subfields of the hippocampus of the mice in the AAV-PGC-1α + RN group were significantly decreased compared with those in the AAV-GFP + RN group. After silencing of PGC-1α in the hippocampus of mice, running exercise could not restore the activity of PV^+^ interneurons in the CA3 subfield. In addition, the cFos^+^/PV^+^ cells in the DG subfield of the hippocampus showed no significant change among the 4 groups after virus injection and running exercise. However, Schoenfeld et al. found that 6 weeks of running decreases the anxiety-like behavior of mice and increases the proportion of PV^+^/cFos^+^ double-labeled cells in the ventral DG subfield but not in the granule cell layer (gcl) or hilus in the dorsal DG subfield^59^. This finding may explain why the DG subfield showed no change after virus injection in our study. We counted the total number of PV^+^/cFos^+^ double-labeled cells in the whole DG subfield, which might be our limitation. The immunofluorescence result in our study firstly showed that the decrease in the activity of PV^+^ interneurons in the CA3 subfield of hippocampus was due to the lack of PGC-1α, which inhibited the effect of running exercise, reversing the pathological changes of PV^+^ interneurons. Therefore, we suggested that running exercise could regulate the activity of PV^+^ interneuron through PGC-1α in hippocampus of the mice to reverse depressive-like behaviors.

Our results indicated that running exercise could not restore the numbers of PV^+^ interneurons after silencing PGC-1α in hippocampus of mice. Previous studies have demonstrated that running exercise has positive effects on the number of PV^+^ interneurons in hippocampus^23,60^. Therefore, is the increase in PV^+^ interneurons in the hippocampus the structural basis of the antidepressant effect of running exercise? We used three-dimensional stereological methods to quantify the total number of PV^+^ interneurons in the DG, CA1 and CA3 subfields of hippocampus of each group of mice after behavioral testing for depressive-like symptoms. The results showed that the numbers of PV^+^ interneurons in the CA1 and CA3 subfields of hippocampus of the mice in the AAV-PGC-1α group were significantly decreased compared to those in the AAV-GFP group, and the numbers of PV^+^ interneurons in the CA1 and CA3 subfields of hippocampus of mice in the AAV-PGC-1α + RN group was significantly decreased compared to those in the AAV-GFP + RN group, but no difference in the numbers of PV^+^ interneurons in DG subfield of the hippocampus among the 4 groups of mice after virus injection and running exercise. We speculated that the reason why running exercise could not improve depressive-like symptoms after PGC-1α gene silencing was because the decrease of PV^+^ interneurons in the CA1 and CA3 caused by the lack of PGC-1α. Therefore, PV^+^ interneurons in hippocampus play an important role in the treatment of depression by running exercise. Combined with the previous results, our findings showed that running exercise could not increase the activity and number of PV^+^ interneurons after PGC-1α silencing, which occurred in the CA3 subfield. Therefore, we speculated that the CA3 subfield is sensitive to the mechanism of running exercise, improving the depressive-like symptoms caused by the lack of PGC-1α. Furthermore, a previous study has indicated that antidepressants could also increase the number of PV^+^ interneurons in all subfields of hippocampus of depressed rats^61^. Therefore, we believe that running exercise may have the same therapeutic target and efficacy as antidepressants to some extent, but running exercise can avoid some of the side effects of antidepressants, which is an advantage in treating depression. Thus, for the first time, we quantified the number of PV^+^ interneurons in mice with regional PGC-1α silencing to explore the therapeutic mechanisms of running exercise on depressive-like symptoms. Our study found that the numbers of PV^+^ interneurons in the hippocampus are closely related to depression and might be an indicator of the illness burdenand treatment response. We demonstrated that running exercise could regulate the activity and numbers of PV^+^ interneurons in the CA3 subfield through PGC-1α in the hippocampus of the mice to reverse depressive-like behaviors, which provided direct support for our initial hypothesis and the antidepressant effects of running exercise.

Depression has become a common mental illness that results in a major burden on society due to its limited cure rate. Although running exercise can relieve depressive-like symptoms, the antidepressant mechanism is still unclear. Therefore, it is important to explore the mechanism of running exercise as a reasonable and effective preventive measure and treatment for depression. In our study, we found that a lack of PGC-1α led to depressive-like symptoms and a decrease in PV^+^ interneurons. More importantly, we demonstrated for the first time that running exercise could regulate PV^+^ interneurons through PGC-1α in the hippocampus of mice to reverse depressive-like behaviors. Our current results might contribute to the exploration of the antidepressant effects of running exercise and the identification of new targets for antidepressant treatment.

## Supplementary information

Supplemental material

## References

[1] Whiteford HA (2013). Global burden of disease attributable to mental and substance use disorders: Findings from the Global Burden of Disease Study 2010. Lancet.

[2] Ferrari AJ (2014). The burden attributable to mental and substance use disorders as risk factors for suicide: Findings from the Global Burden of Disease Study 2010. PLoS ONE.

[3] Cipriani A (2009). Comparative efficacy and acceptability of 12 new-generation antidepressants: a multiple-treatments meta-analysis. Lancet.

[4] Kolar D (2018). Addictive potential of novel treatments for refractory depression and anxiety. Neuropsychiatr. Dis. Treat..

[5] Southwick SM, Vythilingam M, Charney DS (2005). The psychobiology of depression and resilience to stress: implications for prevention and treatment. Annu. Rev. Clin. Psychol..

[6] Schuch FB (2016). Exercise as a treatment for depression: a meta-analysis adjusting for publication bias. J. Psychiatr. Res..

[7] Harvey SB (2018). Exercise and the prevention of depression: results of the HUNT cohort study. Am. J. Psychiatry.

[8] Lee T-H, Kim K, Shin M-S, Kim C-J, Lim B-V (2015). Treadmill exercise alleviates chronic mild stress-induced depression in rats. J. Exerc Rehabil..

[9] Mul JD (2018). Voluntary wheel running promotes resilience to chronic social defeat stress in mice: A role for nucleus accumbens ΔfosB. Neuropsychopharmacology.

[10] Hong Y-P, Lee H-C, Kim H-T (2015). Treadmill exercise after social isolation increases the levels of NGF, BDNF, and synapsin I to induce survival of neurons in the hippocampus, and improves depression-like behavior. J. Exerc. Nutr. Biochem..

[11] Luscher B, Fuchs T (2015). GABAergic control of depression-related brain states. Adv. Pharmacol..

[12] Levinson AJ (2010). Evidence of cortical inhibitory deficits in major depressive disorder. Biol. Psychiatry.

[13] Defelipe J (2013). New insights into the classification and nomenclature of cortical GABAergic interneurons. Nat. Rev. Neurosci..

[14] Tremblay R, Lee S, Rudy B (2016). GABAergic interneurons in the neocortex: from cellular properties to circuits. Neuron.

[15] MacQueen G, Frodl T (2011). The hippocampus in major depression: evidence for the convergence of the bench and bedside in psychiatric research. Mol. Psychiatry.

[16] Frodl T (2002). Hippocampal changes in patients with a first episode of major depression. Am. J. Psychiatry.

[17] Zaletel I, Filipović D, Puškaš N (2016). Chronic stress, hippocampus and parvalbumin-positive interneurons: What do we know so far?. Rev. Neurosci..

[18] CzeH B (2005). Chronic stress decreases the number of parvalbumin-immunoreactive interneurons in the hippocampus: Prevention by treatment with a substance P receptor (NK1) antagonist. Neuropsychopharmacology.

[19] Czéh B (2015). Chronic stress reduces the number of GABAergic interneurons in the adult rat hippocampus, dorsal-ventral and region-specific differences. Hippocampus.

[20] Filipović D, Zlatković J, Gass P, Inta D (2013). The differential effects of acute vs. chronic stress and their combination on hippocampal parvalbumin and inducible heat shock protein 70 expression. Neuroscience.

[21] Csabai D. et al. Electron microscopic analysis of hippocampal axo-somatic synapses in a chronic stress model for depression. *Hippocampus*10.1002/hipo.22650 (2017). 10.1002/hipo.22650 PMC5215622 27571571

[22] Arriaga M, Han EB (2017). Dedicated hippocampal inhibitory networks for locomotion and immobility. J. Neurosci..

[23] Nguyen JCD, Killcross AS, Jenkins TA (2013). Effect of low-intensity treadmill exercise on behavioural measures and hippocampal parvalbumin immunoreactivity in the rat. Behav. Brain Res..

[24] Gomes da Silva S (2010). Physical exercise during the adolescent period of life increases hippocampal parvalbumin expression. Brain Dev..

[25] Villena JA (2015). New insights into PGC-1 coactivators: redefining their role in the regulation of mitochondrial function and beyond. FEBS J..

[26] Marosi K (2012). Long-term exercise treatment reduces oxidative stress in the hippocampus of aging rats. Neuroscience.

[27] Steiner JL, Murphy EA, McClellan JL, Carmichael MD, Davis JM (2011). Exercise training increases mitochondrial biogenesis in the brain. J. Appl. Physiol..

[28] Wrann CD (2013). Exercise induces hippocampal BDNF through a PGC-1α/FNDC5 pathway. Cell Metab..

[29] Belviranlı M, Okudan N (2018). Exercise training protects against aging-induced cognitive dysfunction via activation of the hippocampal PGC-1α/FNDC5/BDNF pathway. NeuroMolecular Med..

[30] Lucas EK (2010). Parvalbumin deficiency and GABAergic dysfunction in mice lacking PGC-1α. J. Neurosci..

[31] Lucas EK (2014). PGC-1α provides a transcriptional framework for synchronous neurotransmitter release from parvalbumin-positive interneurons. J. Neurosci..

[32] Yohn NL, Blendy JA (2017). Adolescent chronic unpredictable stress exposure is a sensitive window for long-term changes in adult behavior in mice. Neuropsychopharmacology.

[33] Logan RW (2015). Chronic stress induces brain region-specific alterations of molecular rhythms that correlate with depression-like behavior in mice. Biol. Psychiatry.

[34] Zhang L (2020). Four-month treadmill exercise prevents the decline in spatial learning and memory abilities and the loss of spinophilin-immunoreactive puncta in the hippocampus of APP/PS1 transgenic mice. Neurobiol. Dis..

[35] Willner P, Towell A, Sampson D, Sophokleous S, Muscat R (1987). Reduction of sucrose preference by chronic unpredictable mild stress, and its restoration by a tricyclic antidepressant. Psychopharmacology (Berl.).

[36] Petit-Demouliere B, Chenu F, Bourin M (2005). Forced swimming test in mice: a review of antidepressant activity. Psychopharmacology (Berl.).

[37] Cryan JF, Mombereau C, Vassout A (2005). The tail suspension test as a model for assessing antidepressant activity: Review of pharmacological and genetic studies in mice. Neurosci. Biobehav. Rev..

[38] Wang J (2020). The effects of fluoxetine on oligodendrocytes in the hippocampus of chronic unpredictable stress-induced depressed model rats. J. Comp. Neurol..

[39] Herrera DG, Robertson HA (1996). Activation of c-fos in the brain. Prog. Neurobiol..

[40] Kovács KJ (1998). c-Fos as a transcription factor: a stressful (re)view from a functional map. Neurochem. Int..

[41] Travis S (2015). Dentate gyrus volume and memory performance in major depressive disorder. J. Affect Disord..

[42] Leal SL, Noche JA, Murray EA, Yassa MA (2017). Disruption of amygdala–entorhinal–hippocampal network in late-life depression. Hippocampus.

[43] Joëls M, Krugers H, Karst H (2007). Stress-induced changes in hippocampal function. Prog. Brain Res..

[44] Daugherty AM, Bender AR, Yuan P, Raz N (2016). Changes in search path complexity and length during learning of a virtual water maze: age differences and differential associations with hippocampal subfield volumes. Cereb. Cortex.

[45] Wang X, Zhang D, Lu XY (2015). Dentate gyrus-CA3 glutamate release/NMDA transmission mediates behavioral despair and antidepressant-like responses to leptin. Mol. Psychiatry.

[46] Daugherty AM, Flinn R, Ofen N (2017). Hippocampal CA3-dentate gyrus volume uniquely linked to improvement in associative memory from childhood to adulthood. Neuroimage.

[47] Wang Y, Xu Y, Sheng H, Ni X, Lu J (2016). Exercise amelioration of depression-like behavior in OVX mice is associated with suppression of NLRP3 inflammasome activation in hippocampus. Behav. Brain Res..

[48] Schwanhüusser B (2011). Global quantification of mammalian gene expression control. Nature.

[49] Azimifar SB, Nagaraj N, Cox J, Mann M (2014). Cell-type-resolved quantitative proteomics of murine liver. Cell Metab..

[50] Marguerat S (2012). Quantitative analysis of fission yeast transcriptomes and proteomes in proliferating and quiescent cells. Cell.

[51] Jovanovic M (2015). Dynamic profiling of the protein life cycle in response to pathogens. Science.

[52] Huntley MA (2020). Genome-wide analysis of differential gene expression and splicing in excitatory neurons and interneuron subtypes. J. Neurosci..

[53] Filice F, Vörckel KJ, Sungur AÖ, Wöhr M, Schwaller B (2016). Reduction in parvalbumin expression not loss of the parvalbumin-expressing GABA interneuron subpopulation in genetic parvalbumin and shank mouse models of autism. Mol. Brain.

[54] Soghomonian JJ, Martin DL (1998). Two isoforms of glutamate decarboxylase: Why?. Trends Pharmacol. Sci..

[55] Cardenas A, Blanca M, Dimitrov E (2019). Persistent pain intensifies recall of consolidated fear memories. Neurobiol. Stress.

[56] Hauser MJ, Isbrandt D, Roeper J (2017). Disturbances of novel object exploration and recognition in a chronic ketamine mouse model of schizophrenia. Behav. Brain Res..

[57] Mumtaz F, Khan MI, Zubair M, Dehpour AR (2018). Neurobiology and consequences of social isolation stress in animal model—a comprehensive review. Biomed. Pharmacother..

[58] Chen CC, Lu J, Yang R, Ding JB, Zuo Y (2018). Selective activation of parvalbumin interneurons prevents stress-induced synapse loss and perceptual defects. Mol. Psychiatry.

[59] Schoenfeld TJ, Rada P, Pieruzzini PR, Hsueh B, Gould E (2013). Physical exercise prevents stress-induced activation of granule neurons and enhances local inhibitory mechanisms in the dentate gyrus. J. Neurosci..

[60] Arida RM (2007). Effects of different types of physical exercise on the staining of parvalbumin-positive neurons in the hippocampal formation of rats with epilepsy. Prog. Neuro-Psychopharmacol. Psychiatry.

[61] Ivana P (2019). Tianeptine antagonizes the reduction of PV+ and GAD67 cells number in dorsal hippocampus of socially isolated rats. Prog. Neuro-Psychopharmacol. Biol. Psychiatry.

