# Supplementary material for: Hippocampal PGC-1α-mediated positive effects on parvalbumin interneurons are required for the antidepressant effects of running exercise
Source: Transl Psychiatry. 2021 Apr 15;11:222. doi: 10.1038/s41398-021-01339-1 (PMC8050070; doi:10.1038/s41398-021-01339-1)
Supplement: Supplementary file 1 — Supplemental material [file 41398_2021_1339_MOESM1_ESM.docx]

**Treadmill running**

Mice in the CUS + Running group, AAV-GFP + RN group and AAV-PGC-1α + RN group were scheduled for treadmill running using a horizontalmotorized treadmill for 5 days per week, 10 min per day. The mice ran at a speed of 5 m/min during the first week, 8 m/min during the second week, and 10 m/min during the third week and maintained the speed for the last week.

**Behavioral tests**

**Sucrose preference test (SPT)**

The SPT is an important assay used to detect anhedonia during CUS model establishment. During the test, each mouse was housed in a single cage with adequate food and water. Mice were presented with two bottles: one bottle was filled with sucrose solution (1%), and the other bottle was filled with water. The bottle containing the sucrose solution was randomly placed on the left or right side of the compartment. The weight of water and sucrose consumed was measured by weighing the bottles. The preference for sucrose was calculated as the percentage of the consumed sucrose-containing solution relative to the total amount of liquid intake over 24 h. After the stress was applied, the SPT was conducted on each mouse at a fixed time each week.

**Forced swimming test (FST)**

FST remains one of the most commonly used tools to test depressive-like symptoms. Mice were placed in a transparent glass cylindrical vessel 20 cm in diameter and 30 cm tall filled to a depth of 20 cm with 22 ± 1 °C water. Immobility time was recorded as the time a mouse floats upright without struggling and only slightly moves its head out of the water. The duration of immobility was recorded during the last 3 min of the total 5 min, which indicated a depressive state.

**Tail suspension test (TST)**

The TST is one of the most widely used tests for assessing depression-like activity in mice. Mice were hung 15 cm above the ground by the tip of the tail, which wastied to the bracket. Immobility was defined when the mouse was hung passively and completely suspended. The duration of immobility was recorded during the last 3 min of the total 5 min, which indicated a depressive state.

**Tissue preparation**

After treadmill running intervention, 5 mice in each group were randomly selected from each group and used for stereological analyses. The mice were injected intraperitoneally with 1% pentobarbital sodium (0.4 ml/100 g) and then perfusion-fixed with 4% paraformaldehyde. After each animal was perfusion-fixed, the cerebrum, meninges, cerebellum and brain stem were removed. The brain was divided into the left and right hemispheres. One hemisphere was randomly selected to be cut into 50-μm-thick serial sections with a cryostatmicrotome (CM1860, Leica). From the sections containing the hippocampus, every 10th section was sampled in a systematic random manner with 6 sections per series on average. In the end, 10 sets of sampled sections were acquired.

**Immunohistochemistry and stereological cell counting**

Two separate sets of serial sections containing the hippocampus from every mouse were chosen and immunoreacted with mouse anti-PV antibody (Sigma, USA) for stereological analyses of the total numbers of PV^+^ interneurons. The immunohistochemistry method and estimation of the number of PV^+^ interneuronswere conducted based on a previous study.

**Immunofluorescence and analysis**

A set of six sampled sections on average from every mouse were labeled with PV (Sigma, USA) antibody and cFos antibody (Abcam, USA) against the active PV^+^ interneuron. Laser scanning confocal microscopy (A1R, Nikon) was used to scan the hippocampus and carry out two-dimensional splicing to compose pictures of the whole hippocampus of every section. Finally, the areas of different subfields of the hippocampus in each picture were measured by Photoshop CC 2019. Quantification of PV/cFos cells in different subfields of the hippocampus in each picture was performed using NIS-Elements viewer 4.2.

**Quantitative real-time PCR**

Total RNA was isolated from the mouse hippocampus using reagent (Promega, China) according to the manufacturer’s instructions. A reverse transcription kit (TaKaRa, China) was applied to perform the reverse transcription experiments. Quantitative real-time PCR was performed using gene-specific primers and SYBR Premix Ex Taq (Bio-Rad, USA). Oligonucleotide primers specific for mice are listed as follows:

PGC-1α-f: TGATGTGAATGACTTGGATACAGACA

PGC-1α-r: GCTCATTGTTGTACTGGTTGGATATG

PV-f:CAGACTCCTTCGACCACAAA

PV-r:GCCACTTTTGTCTTTGTCCA

GAD67-f:CACAGGTCACCCTCGATTTTT

GAD67-r:ACCATCCAACGATCTCTCTCATC

GAD65-f:TCCGGCTTTTGGTCCTTCG

GAD65-r:ATGCCGCCCGTGAACTTTT

Relative mRNA expression levels were analyzed using the 2^−ΔΔCt^ method and normalized to the β-actin ribosomal RNA levels.

**Western blotting and enzyme-linked immunosorbent assays (ELISAs)**

Mice were randomly chosen and anesthetized with sodium pentobarbital, hippocampal tissue was rapidly removed and homogenized, and proteins were extracted using RIPA lysis buffer with 1% PMSF solution (Beyotime Biotechnology, China). After protein determination using a BCA kit (Beyotime Biotechnology, China), sodium dodecyl sulfate-polyacrylamide gel electrophoresis and western blotting were carried out to detect the protein levels of PV, GAD65 (anti-GAD65 antibody, Cell Signaling Technology Inc, USA) and GAD67(anti-GAD67 antibody, Sigma, USA). Quantification of band intensity was performed using Image Lab software (version 5.2.1). The related protein levels detection has been repeated three times. Another part of the protein solution was used to detect the protein level of PGC-1α in the hippocampus of mice by ELISAs.

**Statistical analyses**

All data are expressed as the mean ± standard deviation (SD). The statistical analyses were conducted using SPSS 23.0 statistical software. The Shapiro-Wilk test was used to evaluate whether the data were normally distributed. The Levene’s test was used to evaluate whether the variances were similar among the groups. Student’s t-test was used to compare the results between two groups. One-way ANOVA followed by LSD posthoc test was used to compare the results among the three or four groups. The level of statistical significance was set at *p* < 0.05.
